# Analysis of the Effect of Base Station Motion on Underwater Handovers for Base-Station-Based Underwater Wireless Acoustic Networks

**DOI:** 10.3390/s24123797

**Published:** 2024-06-12

**Authors:** Changho Yun, Yong-Ju Kwon

**Affiliations:** Korea Research Institute of Ships & Ocean Engineering (KRISO), Daejeon 34103, Republic of Korea; kwonyj@kriso.re.kr

**Keywords:** acoustic communication, AUV, BS, buoy, B-UWAN, handover, motion, QoS, service, underwater

## Abstract

In base-station-based underwater wireless acoustic networks (B-UWANs), effective handover mechanisms are necessary to ensure seamless data services for mobile nodes such as autonomous underwater vehicles (AUVs). Unlike terrestrial base stations (BSs), moored buoy BSs in B-UWANs experience motion responses due to wave loads under environmental conditions, posing unique challenges to the handover process. This study examines how BS motion affects handover decision errors, which arise when AUVs incorrectly initiate handovers to unintended BSs due to BS motion. By utilizing the AUV–BS distance as a handover triggering parameter, our analysis reveals a significant increase in decision errors within the overlapping regions when both the current and target BSs are in motion, especially when moving in the same direction. In addition, these errors intensify with the magnitude of BS motion and are exacerbated by smaller BS network radii. Based on these simulation results, we present an analytical framework that not only measures the influence of BS motion on the AUV–BS distance but also provides strategic insights for refining underwater handover protocols, thereby enhancing operational reliability and service continuity in B-UWANs.

## 1. Introduction

Today, the use of mobile nodes such as autonomous underwater vehicles (AUVs) for underwater exploration and cluster searches is increasing [1,2,3]. To effectively coordinate AUVs involved in underwater tasks and transmit collected data to the surface station, an underwater wireless communication network is established by employing floating structures such as buoys [4,5,6]. Acoustic communication, preferred for its reliability in transmitting data over longer distances (ranging from hundreds of meters to tens of kilometers), is the main method employed, as opposed to radio frequency (RF) and optical communications [7,8].

To extend network coverage beyond the communication range of a single buoy, deploying multiple buoys on the water surface allows AUVs to move from one buoy network to another. Buoys, serving as base stations (BSs) akin to terrestrial cellular networks, provide AUVs with network services, with such networks defined as BS-based underwater wireless acoustic networks (B-UWANs). Existing communication and network technologies for B-UWANs have been well developed, with established international standards [9,10,11].

The movement of an AUV between cells may result in temporary channel disconnections or data transmission interruptions. Furthermore, underwater acoustic communication often encounters frequent transmission failures caused by various interferences (e.g., vessel noises, sonar, or dolphins) and environmental conditions, including currents and sea temperature variations [12,13]. Joining a new network under these environments can result in increased network overhead, transmission delays, and extended service unavailability. Therefore, it is necessary to establish measures that guarantee continuous quality of service (QoS), highlighting the need for integrating handover technology within B-UWANs [14].

While studies targeting underwater networks have mainly focused on fixed network topologies, such as underwater acoustic sensor networks, recent trends have shifted towards exploring mobile underwater communication networks, with a particular emphasis on media access control and routing technologies [15,16,17,18,19,20,21]. A review of several studies concerning underwater handover over the past five years indicates that this field is still in its early stages, despite its necessity. Noteworthy research includes machine learning-based handover prediction [22,23], along with the design of handover technologies using underwater RF or optical communication [24,25]. In addition, studies have proposed methods for tracking and predicting the paths of mobile nodes in underwater cellular networks to enhance handover performance [26], as well as addressing the necessity of handover in underwater Internet of Things (IoT) environments [27].

As with the existing handover technologies, underwater handover is also broadly categorized into horizontal and vertical handovers [28,29]. However, underwater communication candidates other than acoustic communication (e.g., RF and optical communications) pose challenges in AUV-based networks characterized by relatively high mobility, attributable to severe communication losses and distance constraints. Accordingly, handover technology for B-UWANs primarily focuses on horizontal handover using exclusively acoustic communication. In contrast to terrestrial handover technology, the development of underwater handover needs to consider the following distinctive factors:

- Since the underwater acoustic frequency band is an open spectrum, it is strongly susceptible to a variety of interferences. Most of them are uncontrollable, with unpredictable locations, operating frequencies, times of occurrence, and durations [30,31,32]. Consequently, frequent communication failures during handovers can result in poor spectrum utilization, high energy consumption, and increasing message overheads.
- The propagation delay of approximately 1 s for a 1.5-km transmission using underwater acoustic signals can significantly boost handover delay [33]. In particular, prevalent message exchanges during handover execution can increase handover delay, leading to handover timeouts.
- Unlike terrestrial environments, where the location of a BS is stationary, buoys floating on the water surface can have variable positions over time. The phenomenon of a floating BS exhibiting mobility is referred to as “BS motion” and is caused by maritime environmental factors, including winds, waves, and currents [34,35], as well as the methods employed for maintaining its position, such as mooring or anchoring [36,37]. The change in the position of a BS over time due to BS motion is referred to as “BS motion response”. It is expected that the BS motion response can have a substantial impact on the triggering of handovers.

The first two factors are often considered to be intrinsic limitations of underwater acoustic communication; it can be intuitively predicted that prevalent communication failures and long propagation delays during handovers can impair the performance of overall handover operations [38]. However, there is a lack of research investigating how the BS motion can affect underwater handovers. Thus, our focus in this paper is directed towards examining the influence of BS motion on underwater handovers.

**Figure 1:**
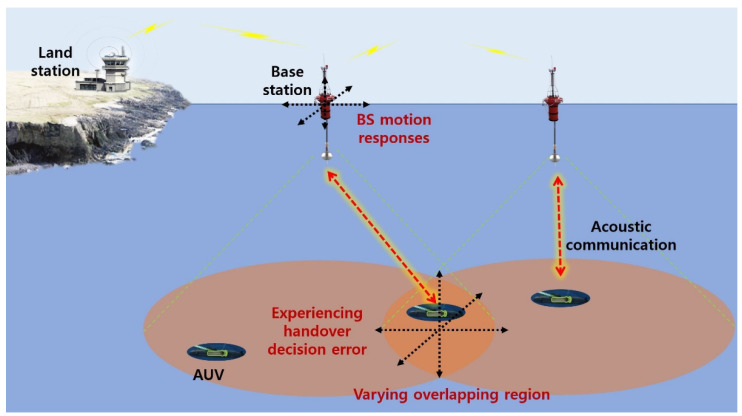
BS motion responses and varying overlapping region due to BS motion.

As depicted in Figure 1, the BS motion can also introduce variability in the overlapping regions of cells. This randomness in the overlapping regions can influence the network connectivity status between the current BS–AUV pair and the target BS–AUV pair. From a handover perspective, frequent triggering events can lead to a handover ping-pong effect. This situation consumes limited resources in underwater acoustic communication, thereby causing network inefficiency and prolonged service disruptions.

To the best of our knowledge, no research has been conducted to investigate the impact of BS motion on underwater handovers; this paper aims to explore the influence of BS motion responses on handover decisions. To do this, we consider the AUV–BS distance as a handover decision criterion [39,40]. This is because the variability in the BS’s location directly leads to fluctuations in the distance between the AUV and BS. Consequently, this variation affects the strength of the signals received from either the AUV or the BS over time, as signal strength decreases with increasing distance [41]. In addition, fluctuations in signal strength induced by varying AUV–BS distance impact the network connectivity of the AUV to the BS. This unstable network connectivity could ultimately induce errors in deciding whether to execute a handover, potentially resulting in frequent handover triggering. Therefore, the analysis of the handover decision errors caused by the BS motion considering the AUV–BS distance is necessary to develop handover strategies for B-UWANs.

This study investigates handover decision errors caused by BS motion, and the assessment is conducted on the basis of the following BS numerical modeling and simulation approaches:

- BS numerical modeling: Generally, BS motion can be calculated via numerical modeling of the buoy and mooring systems in environmental conditions [42]. Rather than considering environmental conditions at a specific location, BS motion is targeted for broad applications under general conditions. To do this, we assume that the BS motion is induced by wave loads with respect to World Meteorological Organization (WMO) sea state codes, as specified in Section 3 [43,44].
- Simulation methods: We analyze the AUV–BS distance by varying the direction of BS motion. This is because the likelihood of the AUV making an incorrect handover decision can increase or decrease depending on the combination of the movement direction of the AUV and the motion direction of the current and target BSs. Furthermore, we check how the WMO sea state code implying the magnitude of BS motion and the BS network radius associated with the overlapping region may result in handover decision errors. Based on the overall simulation results, we derive a distinctive parameter that indicates the variability in the AUV–BS distance caused by BS motion.
- Considering the analysis, we propose several handover considerations, including the influence of BS motion on the AUV–BS distance, as well as how to adaptively respond to the variability in the location of a BS. In addition, we recommend strategies for setting the handover triggering time and configuring the network radius to effectively respond to BS motion.

This paper stands out as a pioneering analysis of the impact of BS motion on handover decisions in B-UWANs; it also introduces an indicative parameter examining the extent of the impact that BS motion has on handover decision errors. Furthermore, the proposed handover considerations in response to BS motion can aid in designing handover protocols for B-UWANs.

The remainder of this paper is structured as follows: Section 2 details the system model considering underwater handover for B-UWANs; Section 3 describes the numerical modeling of a buoy-structured BS; in Section 4, we explain the conditions and assumptions for the simulations, as well as providing an analysis and summary of the simulation results; finally, we conclude this study in Section 5.

## 2. System Model

In this section, we present a system model, and the related parameters are defined as shown in Table 1.

### 2.1. Position and Mobility Description

**Figure 2:**
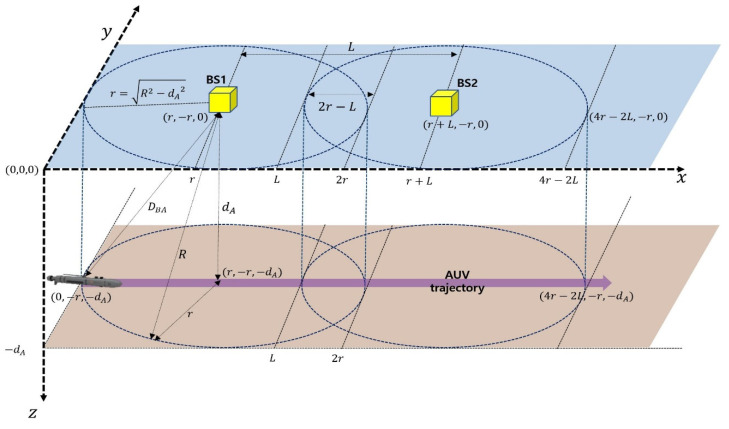
A system model for investigating the impact of BS motion on underwater handovers.

As illustrated in Figure 2, BS1 and BS2 are denoted as the BS to which an AUV is currently connected and the new BS to which the AUV will move through handover (i.e., the target BS), respectively. The communication ranges of the two BSs are overlapped, leading to handover execution when the AUV moves through the overlapping region. The positions and motion of the two BSs are modeled as follows:

- The positions of BS1 and BS2 are represented in an x-y-z coordinate system. As shown in Figure 2, their initial positions are expressed as (r,−r,0) and (r+L,−r,0), respectively.
- The positions of the BSs vary over time along the x-, y-, and z-axes due to BS motion.
- The BS has a buoy-shaped structure and is moored to the seafloor with the help of the mooring line.
- The two BSs are separated by the distance of L on the water surface, and the width of the overlapping region (i.e., 2r−L) is determined based on the overlapping rate (δ). As illustrated in Figure 2, δ is defined as $\frac{2r-L}{2r}$. According to the value of L, δ is given as $\delta =\left\{\begin{array}{cc} 0, & L=2r\\ 0.5, & L=r\\ 1.0, & L=0 \end{array}\right.$. For instance, δ=0 implies that the two cells are not overlapped at all. In this paper, while the overlapping rate is simply determined by the width of the overlapping region, it is possible to define the overlapping rate more precisely by considering the overlapping region in three dimensions.
- The AUV generally moves underwater with respect to a scheduled path plan with a consistent direction and speed [45]. By considering these characteristics of the AUV, we can define $T_{O}$, i.e., the time the AUV spends in the overlapping region. By using the moving speed of the AUV (i.e., $v_{A}$) and the width of the overlapping region (i.e., 2r−L), $T_{O}$ can be defined as $\frac{2r-L}{v_{A}}=\frac{2r\delta }{v_{A}}$. This parameter can be utilized to determine the handover expiration time concerning AUV movement.
- The location and mobility of an AUV are modeled as follows:
- The position of an AUV is also expressed in an x-y-z coordinate system. As depicted in Figure 2, its initial position is given as $\left(0,-r,-d_{A}\right)$, which is at the network edge of BS1.
- The AUV is programmed to move from the network edge of BS1 to that of BS2 (in the positive x-axis direction) at a constant speed. In the system model, the AUV is considered to move along a planned one-way path from the edge of BS1 to the edge of BS2.

### 2.2. Communication and Networking

We consider a three-dimensional B-UWAN, as illustrated in Figure 2. A B-UWAN is normally composed of multiple BSs on the water surface and several AUVs. Each BS communicates with an AUV that is currently located in its cell. AUVs search the target area and obtain data. Then, they transmit their sensed data to their BS. A BS also acts as an underwater wireless backhaul, because the BS transfers all of the gathered data from the AUVs to a land station, as shown in Figure 1. To do this, there are two communication links in the BS: one is the underwater acoustic link between the BS and an AUV, and the other is the maritime wireless link between the BS and the land station. The maritime wireless communication can be exemplified as LTE, VHF, Wi-Fi, or satellite communication [46]. In this paper, we consider two BSs (i.e., BS1 and BS2) and only one AUV to describe the situation in which handover occurs.

All of the nodes of a B-UWAN are equipped with a digital acoustic communication module that provides the following common characteristics:

- Multiple channels are available. One is a common control channel used for control signaling, such as channel access or handover. Others are data channels purely employed for data transmission. This ensures sufficient bandwidth for the transmission of underwater data sensed by an AUV in challenging underwater conditions.
- The communication between a BS and an AUV can be half-duplex or full-duplex. The communication method is determined considering operational scenarios, along with the volume and transmission frequency of control and data messages.
- An AUV and a BS can communicate with one another within their communication range by employing an omnidirectional antenna. This is because it is challenging to align antenna directivity between fast-moving AUVs and unpredictably moving BSs.
- It is assumed that any AUV can connect to its current BS directly by sending signals with the maximum power.

The networking of a B-UWAN is described as follows:

- A remotely operated AUV needs to periodically transmit control signals such as its current survival status and underwater location information to its current BS [47,48]. In addition, depending on the situation, the AUV may need to transmit large volumes of sensed data to the BS in burst mode. To do this, several control and data messages can be exchanged between the BS and the AUV.
- For a common control channel, the time domain for uplink and downlink transmission needs to be efficiently divided. For uplink transmission of control signaling, a time-based scheduling approach is employed, enabling AUVs to transmit their messages efficiently and without collision. For data channels, an adequate channel access scheme should be employed in order to not only avoid collisions but also enhance spectrum efficiency.
- As any AUV can transmit its acoustic signals to its current BS in one hop, no routing strategies are employed.
- The AUV transitions to the network area of a different BS. To maintain continuous data service for the AUV in the network of a new BS, proper handover procedures are implemented. Detailed descriptions of these handover scenarios are provided in Section 2.3.

### 2.3. Handover Scenario

The characteristics of the handover for a B-UWAN are outlined as follows:

- The handover for a B-UWAN is horizontal, indicating that the transition occurs within an acoustic communication network with the same frequency band, modulation and coding scheme, and network configurations.
- Handover triggering is performed by an AUV. In other words, when the handover conditions are satisfied, the AUV initiates a handover request to the BS, negotiating to maintain the continuity of its service between the current BS and the target BS.
- An AUV moving along a planned trajectory can predict its current location with the help of location-tracking equipment, such as an ultra-short baseline. Unlike terrestrial cellular networks, where node movement is random, in a B-UWAN, it is unnecessary to consider handovers in every region within a cell. Instead, handovers can be considered only when the AUV is located in the overlapping region. This characteristic stands out, as it aptly captures the distinctive attributes of the AUV, and, when effectively leveraged, it substantially enhances the efficiency of handover by minimizing associated overheads.

When an AUV moves from BS1 to BS2, the handover processes for a B-UWAN can be briefly described as follows:

- Handover is considered when an AUV approaches the overlapping region, receiving acoustic signals not only from BS1 but also from BS2.
- When the AUV is present in the overlapping region and the handover conditions are met, it requests handover from BS1. As mentioned above, the AUV–BS distance is considered as a handover decision parameter; it is determined by using the transmission time information of messages sent by the BS and the time information received by the AUV, along with the propagation speed of acoustic signals.
- Upon receiving a handover request from the AUV, BS1 initiates a handover negotiation with BS2 using available maritime communication. During this process, BS1 communicates the QoS requirements of the AUV, and BS2 allocates resources that satisfy the QoS of the AUV.
- BS1 notifies the AUV of a successful handover and, simultaneously, terminates the network connection to the AUV. As the AUV enters the network of BS2, the handover process concludes.
- Given the challenging conditions of underwater communication, it is common to experience frequent transmission failures during the handover process. To mitigate this, it is necessary to configure parameters such as handover timeout and retry attempts, effectively preventing unnecessary resource consumption in the handover procedure.

The scenarios described in Section 2.3 represent one simple example of the handover processes tailored for a B-UWAN. Scenarios for underwater handovers can be extended or include more complex procedures.

## 3. Environmental Conditions and Buoy Modeling

In this section, a buoy-shaped BS is numerically modeled to investigate the effects of BS motion on underwater handovers. For the analysis, an example of a moored ocean buoy is considered in the deep water. Here, “buoy” and “BS” are used interchangeably and refer to the same entity.

### 3.1. Environmental Loads and Conditions

An ocean buoy experiences various environmental forces, including waves, winds, and currents [32,33]. This study investigated how BS motion affects wave loads across different sea conditions. We assumed that the wind and current velocities remain constant across sea states, with wind speed set at 10 m/s and current speed around 1 m/s near the water surface. Generally, wind and current loads are considered to be static loads, and this approach was applied in this simulation. Different wind and current loads cause the position of the buoy to change. However, in the case of multiple buoys, assuming that the loads between buoys are the same, the change in their relative position is not significant. Wave forces are dynamic and are calculated using Morison’s equation, which separates the drag and inertia components [49]. To ensure wide applicability, we utilized the wave spectrum defined by the WMO ranging from sea state 3 (SS3) to sea state 8 (SS8), which was originally developed for assessing ship performance in various environmental conditions. The WMO sea state codes are further described in Table A2 in Appendix B. In addition, we incorporated the Joint North Sea Wave Project (JONSWAP) spectrum into our analysis, with a peak enhancement factor of 3.3 [50].

### 3.2. Numerical Analysis with Buoy Modeling

**Figure 3:**
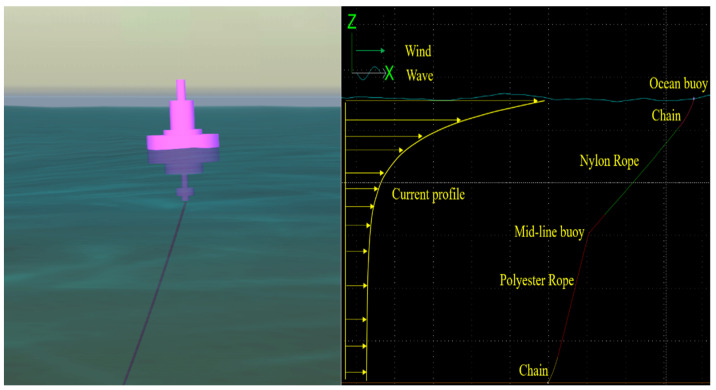
Numerical modeling for a buoy (**left**) and a model including a mooring line (**right**) under sea state 8.

The numerical analysis was conducted using OrcaFlex 11.2b, a commercial software package operated in Windows that incorporates buoy geometry and Morison force calculation [51]. In this analysis, we utilized a 4-ton buoy with a maximum diameter of 2.8 m, as illustrated in the left panel in Figure 3. The buoy is moored using a single mooring line consisting of chain–nylon–polyester–chain, as shown in the right panel in Figure 3. A mid-line buoy is positioned at the midpoint of the mooring line, approximately 340 m away from the ocean buoy, where the current velocity is noticeably reduced. Due to the numerical modeling of the buoy, the x, y, z coordinates of the ocean buoy are not fixed but instead vary over time, either increasing or decreasing. These time-dependent ocean buoy responses, corresponding to different sea state conditions, are utilized as simulation conditions in Section 4.

## 4. Performance Analysis

In this section, we analyze the AUV–BS distance by varying the direction and magnitude of BS motion in order to investigate its effect on handover decision errors. For this purpose, the parameters related to AUV–BS distance are defined in Table 2. Next, we describe the simulation conditions and assumptions. Then, we analyze the overall simulation results and provide several handover considerations for B-UWANs.

### 4.1. Parameter Definition

As mentioned in the Introduction, this study aims to explore the influence of BS motion on handover decisions. For this purpose, we consider the AUV–BS distance as a handover decision criterion, which is denoted as $D_{BA}$, as depicted in Figure 2. This is because the initial positions of the BSs, as described in Section 2.1, experience changes across the x-, y-, and z-axes, which can be attributed to the dynamics of BS motion. This variation affects the network connectivity of the AUV to the BS and, consequently, induces handover decision errors.

In Section 2, we define BS1 and BS2 as the current serving BS and the target BS for handover, respectively. Initially, we derive distance parameters related to the AUV and BS1. For this purpose, we consider the coordinates of the AUV (defined as $\left(X_{A},Y_{A},Z_{A}\right)$), the coordinates of BS1 when stationary (defined as $\left(X_{F1},Y_{F1},Z_{F1}\right)$), and the coordinates of BS1 when its position changes due to BS motion (defined as ($(X_{M1},Y_{M1},Z_{M1}$)). Then, the distance between the AUV and stationary BS1, $D_{BA1F}$, is expressed as $\sqrt{{\left(X_{F1}-X_{A1}\right)}^{2}+{\left(Y_{F1}-Y_{A1}\right)}^{2}+{\left(Z_{F1}-Z_{A1}\right)}^{2}}$. The distance between BS1 and an AUV where BS1 is nonstationary due to BS motion, $D_{BA1M}$, is also given as $\sqrt{{\left(X_{M1}-X_{A}\right)}^{2}+{\left(Y_{M1}-Y_{A}\right)}^{2}+{\left(Z_{M1}-Z_{A}\right)}^{2}}$. In addition, $D_{BA2F}$, the distance between the AUV stationary BS2, and $D_{BA2M}$, the distance between the AUV and nonstationary BS2, can be similarly determined.

To determine the effect of BS motion, we consider the difference between $D_{BA1F}$ and $D_{BA1M}$ for BS1 (defined as $\Delta D_{BA1}$) and the difference between $D_{BA2F}$ and $D_{BA2M}$ for BS2 (defined as $\Delta D_{BA2}$). Additionally, we compare the distances between the AUV and each of the two BSs in order to check the impact of BS motion on handover. To do this, we consider the difference between $D_{BA1F}$ and $D_{BA2F}$ for the stationary BS (defined as $D_{BA12F}$), as well as the difference between $D_{BA1M}$ and $D_{BA2M}$ for the nonstationary BS (defined as $D_{BA12M}$). Finally, we also denote the difference between $D_{BA12F}$ and $D_{BA12F}$ as $\Delta D_{BA12}$.

Table 2 summarizes the overall parameters related to the AUV–BS distance. Parameters for a stationary BS are marked with an “F” subscript (e.g., $D_{BA1F}$), whereas those reflecting the BS motion use an “M” subscript (e.g., $D_{BA1M}$). The absence of “F” or “M” in a parameter subscript implies that the parameter covers both cases. In addition, the unit for all parameters in Table 2 is meters.

### 4.2. Simulation Assumptions and Conditions

For the simulation, we consider a network scenario consisting of two BSs and one AUV, as depicted in Figure 2. There exists an overlapping region between the network areas of these two BSs, where handover can be conducted. Under this scenario, the simulation considers the following assumptions:

- Underwater acoustic communication can suffer from underwater background noises, which induce severe interferences and, thus, frequent transmission failures. However, to investigate the pure effect of BS motion on the handover decision, it is assumed that the received signal degradation due to background noises is not considered in simulations.
- The AUV moves along its programmed path with a uniform speed and direction.
- The AUV receives sufficient messages from the two BSs to determine the distances to them, and it can calculate the AUV–BS distance based on the transmission and reception timestamps of these messages.
- In the simulation, we only consider a one-way scenario where the AUV moves from the network edge of BS1 to the network edge of BS2, as illustrated in Figure 2.
- We assume buoys floating on the water surface that have variable positions over time caused by diverse maritime environmental conditions, including winds, waves, and currents, as well as the methods employed for maintaining their position, such as mooring or anchoring. This situation can have a substantial impact on the triggering of handovers.

**Figure 4:**
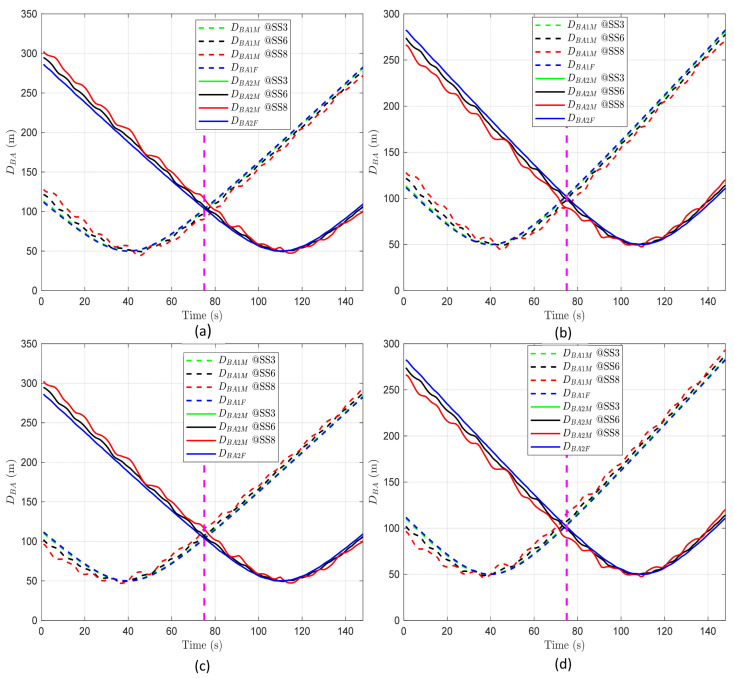
$D_{BA}$ according to time and BS motion. (**a**) BS1 and BS2 move in the same direction as the AUV. (**b**) BS1 moves in the same direction as the AUV, while BS2 moves in the opposite direction. (**c**) BS1 moves in the opposite direction to the AUV, while BS2 moves in the same direction. (**d**) BS1 and BS2 move in the opposite direction to the AUV.

The simulation was executed using MATLAB software (ver. R2023a) under the following conditions:

- In simulations, the AUV–BS distance is primarily derived considering the BS motion response over time. Here, the BS motion response is determined considering various environmental conditions, such as wind and waves, as explained in Section 3. Therefore, by varying the direction and magnitude of BS motion, the BS motion response is derived and applied to the simulations. If the BS moves in the same direction as the AUV, the AUV–BS distance reduces, possibly leading to decision errors, in contrast to a stationary BS. Additionally, the relative motion directions of BS1 and BS2 can also cause errors, since BS motion affects the difference between $D_{BA1}$ and $D_{BA2}$. Consequently, this variability could result in the AUV erroneously selecting a BS, since it aims to connect to the closest network. The direction of BS motion entails four possible combinations based on (1) whether the BS moves in the same direction as the AUV and (2) whether the motions of BS1 and BS2 are directionally the same. We consider these four cases in analyzing the AUV–BS distance. These four cases are described in the caption of Figure 4.
- In addition, the magnitude of BS motion can affect the extent of positional changes in the x, y, and z coordinates; that is, the larger the BS motion, the greater the potential variability in the AUV–BS distance. This paper considers three cases according to the WMO sea state codes. Among the codes defined by the WMO, ranging from 0 to 9, SS3 (sea state code = 3, slight), SS6 (very rough), and SS8 (very high) are considered [43].
- To check the influence of network radius on handover decisions, we consider four network radii of 100, 300, 500, and 1000 m.
- The operating depth of the AUV ($d_{A}$) is set at 50 m.
- As shown in Figure 2, the initial positions of BS1, BS2, and AUV are given as (r,−r,0), (r+L,−r,0), and (0$,-r,{-d}_{A}$), respectively. The straight-line distance between the two BSs on the water surface, denoted as L, is set using the overlap rate value δ and r (i.e., L=(1−δ)×2r) where δ is set to 0.1.
- The moving speed of the AUV is set at 5 knots, where 1 knot is approximately equal to 0.514 m/s.
- In the simulations, we varied the conditions of BS motion direction, magnitude, and network radius. To ascertain the unique impact of each factor on the handover process, the simulations were conducted by altering only one condition at a time.
- All simulation conditions are summarized as shown in Table 3.

The simulations were conducted across two scenarios. In the first scenario, the network radius was kept constant to assess the impact of BS motion, with variations in its direction and magnitude. In the second scenario, with a fixed direction and magnitude of BS motion, the focus shifted to evaluating the effects of network radii. In addition, the simulation was based on BS motion response data derived according to the WMO sea state code, as described in Section 3. This simulation incorporated various practical environments, including buoy mooring modeling, WMO sea state codes, and JONSWAP wave spectrum models, to obtain the BS motion response data. Therefore, unlike traditional network simulations that require numerous trials to obtain an average due to random conditions, this approach has the advantage of not necessitating such an extensive number of simulations.

### 4.3. Simulations Results Corresponding to BS Motion

#### 4.3.1. Analysis of $D_{BA}$

As illustrated in Figure 4, regardless of the direction and magnitude of BS motion, the minimum value of $D_{BA1}$ is approximately 50 m. From the point where $D_{BA1}\;$ reaches its minimum, it decreases before that point and increases afterwards. $D_{BA2}$ exhibits a similar pattern to $D_{BA1}$. The common results of $D_{BA}$ based on the direction of BS motion can be also summarized as follows:

- The distance between an AUV and BS1 varies depending on BS1’s motion relative to the AUV. When BS1 moves in the same direction as the AUV, the distance between them, measured before reaching the closest point (minimum distance), shows that $D_{BA1F}$ is less than $D_{BA1M}$. After surpassing this closest point, if BS1 continues in the same direction, the relationship flips, making $D_{BA1F}$ greater than $D_{BA1M}$, as depicted in Figure 4a. Conversely, if BS1 moves in the opposite direction to the movement of the AUV, the relationship between $D_{BA1F}$ and $D_{BA1M}$ around the closest point reverses compared to when BS1 moves in the same direction as the AUV, as shown in Figure 4b. This behavior demonstrates that the distance between the AUV and BS1 changes notably depending on the direction of BS1’s motion. When they move towards one another, the distance decreases, and when they move away from one another, the distance increases.
- Similarly, for BS2, the relationship between $D_{BA2F}$ and $D_{BA2M}$ also varies at the point of minimum distance, depending on the direction of BS2’s motion relative to the direction of the AUV’s movement, as shown in Figure 4c,d.

As the magnitude of BS motion increases, it can be observed that both $D_{BA1M}$ and $D_{BA2M}$ exhibit greater fluctuations compared to $D_{BA1F}$ and $D_{BA2F}$, as illustrated in all panels in Figure 4. This suggests that the distance between the AUV and the BS becomes more unpredictable and varies more widely when the BS is in motion. Moreover, as the conditions at sea become rougher (indicated by a higher sea state code), the fluctuations in these distances grow even more severe. Hence, the rougher the sea, the more unpredictable the distance between the AUV and the BS becomes, making it harder to maintain consistent communication.

#### 4.3.2. Analysis of $\Delta D_{BA1}$ and $\Delta D_{BA2}$

In this section, we examine the differences in the distances due to BS motion in comparison to the distances from a stationary BS. These differences are represented as $\Delta D_{BA1}$ (=$D_{BA1F}-D_{BA1M}$) for BS1 and $\Delta D_{BA2}$ (=$D_{BA2F}-D_{BA2M}$) for BS2, as defined in Table 2. In the leftmost two columns in Table 4, the designation “F” denotes the condition where the BS’s motion is aligned with the trajectory of an AUV, referred to as the forward direction. Conversely, “R” denotes the reverse direction, indicating that the BS is moving opposite to the path of the AUV. “STD” stands for standard deviation, a measure indicating the extent of variation from the average distance.

The statistical analysis of $\Delta D_{BA1}$ and $\Delta D_{BA2}$ indicates that while the mean values may differ in sign depending on the direction of BS motion, their absolute values remain closely matched. The impact of BS motion on these measurements is pronounced, as the absolute values of the means increase with respect to the sea state code. The standard deviations for $\Delta D_{BA1}$ and $\Delta D_{BA2}$ are nearly the same, irrespective of the BS motion direction, and they increase according to the sea state code. This result shows that the variability in AUV–BS distance is more significantly influenced by the magnitude of BS motion than its direction, emphasizing the substantial effect of the intensity of BS motion on distance fluctuations.

The statistical results indicate an increase in both the mean and standard deviation for $\Delta D_{BA1}$ and $\Delta D_{BA2}$ with a higher sea state code, indicating a direct relation between worsening sea conditions and increased variability in AUV–BS distance due to BS motion. Although the analysis sheds light on the variability introduced by BS motion, determining its specific impact on handover decisions remains complex. This analysis provides insights into the presence of the variability, but it does not offer a definitive conclusion on how it affects the likelihood of handover errors, calling for further investigation to fully understand its implications for handover strategies.

#### 4.3.3. Analysis of $D_{BA12}$

When deciding to execute a handover based on the AUV–BS distance, it is necessary to compare the values of $D_{BA1}$ and $D_{BA2}$; this is relative to the AUV to determine which BS is closer. Accordingly, this analysis involves examining $D_{BA12}$, which enables comparison between $D_{BA1}$ and $D_{BA2}$.

**Figure 5:**
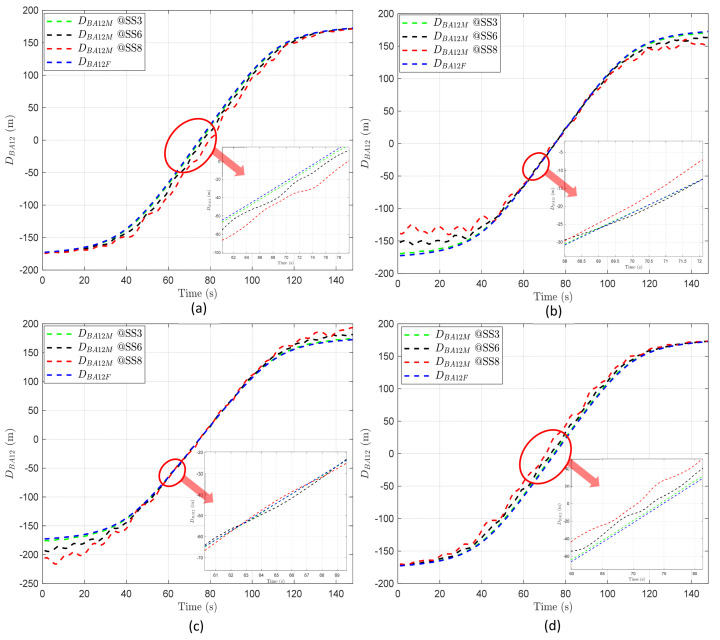
$D_{BA12}$ with respect to time and BS motion when r is 100 m: (**a**) BS1 and BS2 move in the same direction as the AUV. (**b**) BS1 moves in the same direction as the AUV, while BS2 moves in the opposite direction. (**c**) BS1 moves in the opposite direction to the AUV, while BS2 moves in the same direction. (**d**) BS1 and BS2 move in the opposite direction to the AUV.

As shown in all panels in Figure 5, $D_{BA12}$ commonly starts with a negative value, progresses to zero, and subsequently shifts to a positive value as time passes. This consistent behavior occurs irrespective of the direction or magnitude of BS motion, attributed to the scenario wherein the AUV steadily transitions from the coverage area of BS1 to that of BS2.

However, the variability pattern of $D_{BA12}$ shifts based on the BS motion direction. When BS1 and BS2 move in the same direction, the variability spikes in the overlapping region, meaning that the gap between $D_{BA12F}$ and $D_{BA12M}$ widens, as shown in Figure 5a,d. Conversely, when BS1 and BS2 move in opposite directions, the variability heightens near the start and end points of the path of the AUV, as depicted in Figure 5b,c. This demonstrates that, in scenarios where the AUV transitions from the periphery of BS1 to BS2, the instances of increased or decreased variability are influenced by BS motion direction.

As the magnitude of BS motion increases, the variability in $D_{BA12}$ also intensifies. In scenarios where the motion directions of BS1 and BS2 are the same, the difference between $D_{BA12F}$ and $D_{BA12M}$ grows as the BS motion magnitude increases, especially during the time at which the AUV passes through the overlapping region, as illustrated in Figure 5a,d. Conversely, when the motion directions of BS1 and BS2 are opposite, the variability in $D_{BA12}$ increases near the start and end points of the movement of the AUV as the sea state code rises, demonstrating a notable impact of BS motion magnitude in these scenarios, as shown in Figure 5b,c.

#### 4.3.4. Analysis of ${\Delta D}_{BA12}$

The variability in ${\Delta D}_{BA12}$ is determined by conditions such as the direction of AUV movement, the direction of BS motion, and the magnitude of BS motion. Through the analysis of $D_{BA}$, described in Section 4.3.1, it was observed that the $D_{BA}$ value varies over time relative to the direction of BS motion compared to the stationary BS, based on the point where the $D_{BA}$ value reaches its minimum. Building on this result, the analysis of $D_{BA12}$ in Section 4.3.3 indicates that, depending on the motion directions of BS1 and BS2, the $D_{BA12}$ value can increase in the overlapping region or reach its maximum at the network edge.

To statistically examine the variability introduced by BS motion to $D_{BA12}$, we analyzed the mean and standard deviation of ${\Delta D}_{BA12}$ according to the direction and magnitude of BS motion, as presented in Table 5.

Similar to ${\Delta D}_{BA1}$ and ${\Delta D}_{BA2}$, the mean of ${\Delta D}_{BA12}$ also varies between positive and negative values depending on the direction of BS motion, yet the absolute values remain comparably similar. Furthermore, the absolute value of the mean increases as the sea state code rises. The standard deviation of ${\Delta D}_{BA12}$ also maintains nearly identical values regardless of the direction of BS motion, with an increase in standard deviation as the sea state code increases. Based on these results, the mean and standard deviation of ${\Delta D}_{BA12}$ are more influenced by the magnitude of BS motion rather than its direction, consistent with the results in Section 4.3.2.

In the two cases where the direction of motion for BS1 and BS2 is the same, the values of ${\Delta D}_{BA12}$ are equal but have opposite signs. Similarly, in the other two cases, where the direction of motion for BS1 and BS2 differs, the pattern remains the same. Considering this symmetry of ${\Delta D}_{BA12}$ with respect to the direction of BS motion, we can consider $\left|{\Delta D}_{BA12}\right|$ as an indicator of variability due to the effect of BS motion instead of ${\Delta D}_{BA12}$; that is, $\left|{\Delta D}_{BA12}\right|$ can be used to infer when the variability in the AUV–BS distance might become significantly severe.

**Figure 6:**
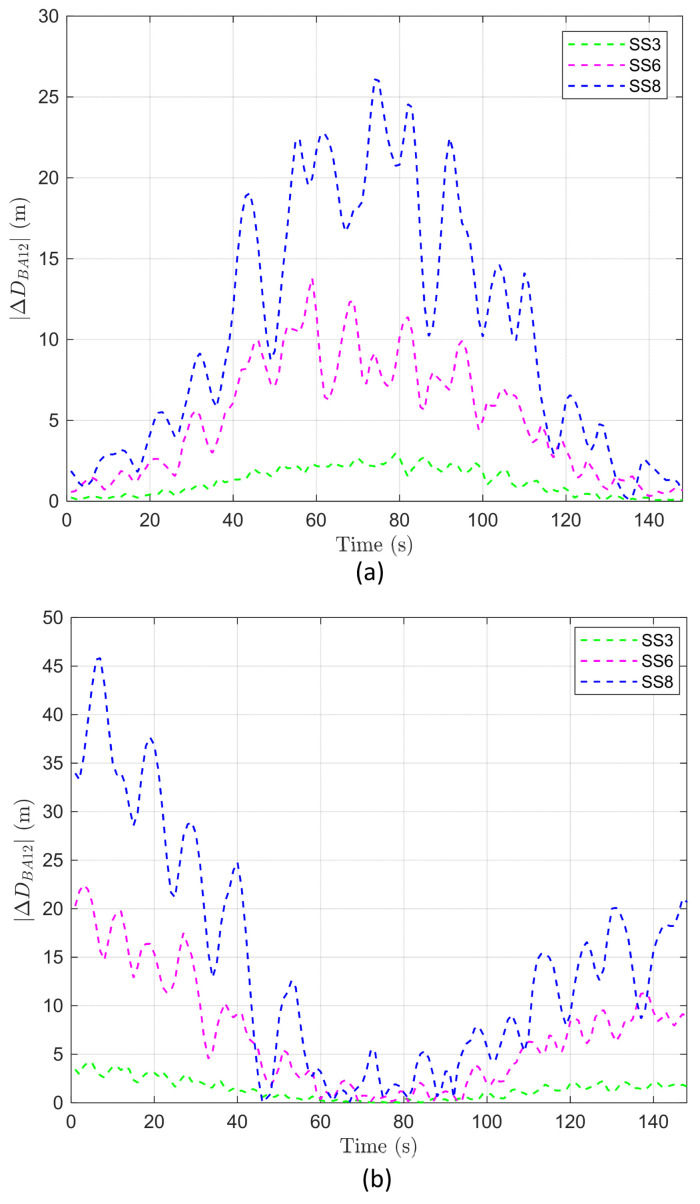
$\left|{\Delta D}_{BA12}\right|$ according to time and BS motion when r is 100 m: (**a**) When the motion of BS1 and BS2 is in the same direction. (**b**) When the motion of BS1 and BS2 is in opposite directions.

As depicted in Figure 6a, in cases where the direction of motion for BS1 and BS2 is the same, the value of $\left|{\Delta D}_{BA12}\right|$ is larger during the period passing through the overlapping region than at the points crossing the edges, and this difference increases as the sea state code rises. In cases where the direction of motion for BS1 and BS2 is opposite, the pattern of $\left|{\Delta D}_{BA12}\right|$ is found to be the exact opposite, as shown in Figure 6b. However, as depicted in Figure 5b,c, when $D_{BA12}$ exceeds 150 m at the network edge, a variability of less than 50 m in $\left|{\Delta D}_{BA12}\right|$ does not affect the handover decision. Conversely, as illustrated in Figure 5a,d, when $D_{BA12}$ is less than 50 m, variations in $\left|{\Delta D}_{BA12}\right|$ below 50 m can lead to erroneous handover decisions. These analytical findings suggest that considering only cases where variability increases in the overlapping region is sufficient.

In addition, the analysis of $\left|{\Delta D}_{BA12}\right|$ allows us to identify the timepoints where the impact of BS motion is most significant, and to infer the extent of variability caused by the direction and magnitude of BS motion. This suggests that $\left|{\Delta D}_{BA12}\right|$ can serve as a measure to evaluate the degree of variability introduced to the AUV–BS distance due to BS motion.

#### 4.3.5. Analysis of Handover Decision Errors Due to BS Motion

As shown in Figure 5a, at the start and end points of the movement of an AUV, the absolute value of $D_{BA12}$ is large. This result means that there is minimal confusion about whether the AUV should connect to BS1 or BS2, essentially indicating that handover is unnecessary at these times. On the other hand, during the period of passing through the overlapping region, the value of $D_{BA12}$ approaches ”0”. This implies that the variability caused by BS motion significantly increases the likelihood of handover decision errors. From these results, it can be inferred that considering the handover decision errors based on the AUV–BS distance is justifiable for the cases where the motions of BS1 and BS2 are in the same direction; that is, as shown in Figure 6a, when the value of $\left|{\Delta D}_{BA12}\right|$ (i.e., variability) is large in the overlapping region, the probability of handover decision errors may also increase.

We further analyzed $D_{BA12}$ according to sea state conditions by zooming into the periods when crossing the overlapping area and near the endpoints in Figure 5a. Comparing the overall effect of BS motion on ${\Delta D}_{BA12}$ throughout the entire time with its effect during the period of crossing the overlapping region yielded the following: for ${\Delta D}_{BA12}$ at SS3, the average variation throughout all points is ~1.18, but it increases to 2.34 during the period of crossing the overlapping region. For ${\Delta D}_{BA12}$ at SS6, the average variation throughout all points is ~5.08, but it increases to 8.38 during the period of crossing the overlapping region. Lastly, for ${\Delta D}_{BA12}$ at SS8, the average variation throughout all points is ~10.35, but it dramatically increases to 21.61 during the period of crossing the overlapping area. This indicates that the period of crossing the overlapping region, where a handover needs to be performed, is more significantly affected by BS motion.

**Figure 7:**
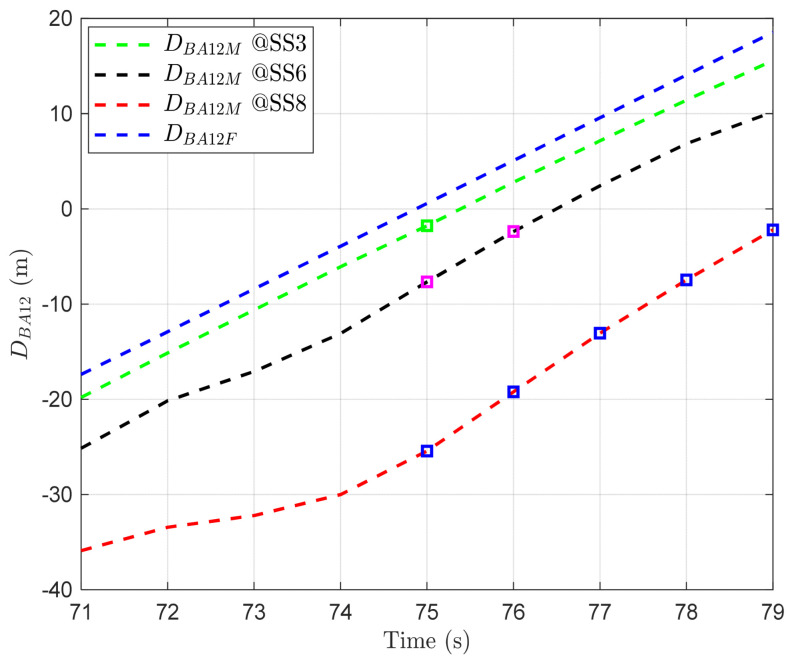
$D_{BA12}$ with respect to time and sea state in the overlapping area when r is 100 m, and BS1 and BS2 move in the same direction as the AUV.

Building on the analysis above, we zoomed into Figure 5a, as illustrated in Figure 7, to examine how often handover decision errors occur during the period of highest $\left|{\Delta D}_{BA12}\right|$ variability, when the motion direction of BS1 and BS2 is the same, specifically in the overlapping region. The squares above the graphs are the points at which errors in distance judgment occur due to BS motion (i.e., when BS1 is judged to be closer, but due to BS motion, BS2 is actually closer, and vice versa). As depicted in Figure 7, handover decision errors occur starting from 75 s in the overlapping region under the given sea state code conditions. This implies that when the motion directions of the two BSs are the same, the AUV may experience periodic handover decision errors upon entering this overlapping region, due to incorrect handover triggering. Moreover, this highlights that as the sea conditions deteriorate, environmental changes such as waves and wind become more severe, thereby exerting a greater influence on communication signals. This causes a mismatch between the expected and actual positions of the BSs, leading to incorrect assessments of the relationship between $D_{BA1}$ and $D_{BA2}$.

The overall results imply that the sea state induces BS motion, which, in turn, increases the variability in AUV–BS distance. It can be observed that when the motions of two BSs are in the same direction, the likelihood of errors in handover decisions increases with the magnitude of BS motion. Specifically, this variability is further amplified in the overlapping region, potentially leading to frequent errors upon determining the timing for handover triggering.

### 4.4. Simulations Results Corresponding to Network Radius

Through the system model in Section 2, it is intuitively known that the network radius of the BS plays an important role in defining the width of the overlapping region. By considering the insights gained from examining the effects of BS motion in Section 4.3, this analysis aims to understand the influence of the BS network radius on handover decision errors, particularly in scenarios where the direction of motion for BS1 and BS2 is the same as the direction of AUV movement.

#### 4.4.1. Analysis of $D_{BA}$

**Figure 8:**
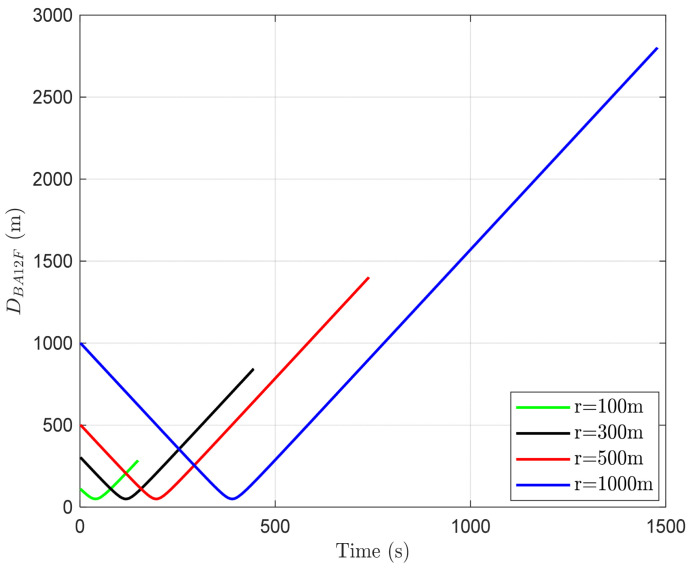
$D_{BA1F}$ according to time and network radii.

For the analysis based on network radius, we consider the distance between the AUV and BS1 when BS1 is stationary, denoted as $D_{BA1F}$. As shown in Figure 8, despite changes in network radii, the minimum value of $D_{BA1F}$ remains approximately 50 m. This is because $D_{BA1F}$ reaches its minimum value when the AUV is aligned perpendicularly with BS1, regardless of the network radius. However, as the network radius increases, both the range of $D_{BA1F}$ and the timepoint when $D_{BA1F}$ reaches its minimum value also increase. This is caused by the fact that the AUV starts from the network edge of BS1, and as the network radius increases, it takes longer to reach the point where the distance to BS1 is minimized. In addition, as the network radius increases, the distance that the AUV travels also increases, resulting in an expansion in the range of $D_{BA1F}$.

Furthermore, an increase in network radius also enlarges the overlapping region. This implies that the time taken to traverse this overlapping region increases as well. The characteristics derived from the analysis of $D_{BA1F}$ based on network radius are not directly influencing factors for handover decision errors; thus, it is essential to analyze $\left|{\Delta D}_{BA12}\right|$, which represents variability.

#### 4.4.2. Analysis of $\left|{\Delta D}_{BA12}\right|$

Even as the network radius changes, the BS motion modeling remains unchanged, meaning the impact of BS motion on AUV–BS distance is maintained. Hence, irrespective of the expansion or contraction of the network radii, the observed pattern remains consistent: variability increases as the AUV traverses the overlapping region when the motion directions of BS1 and BS2 align, and this variability increases with increasing BS motion.

**Figure 9:**
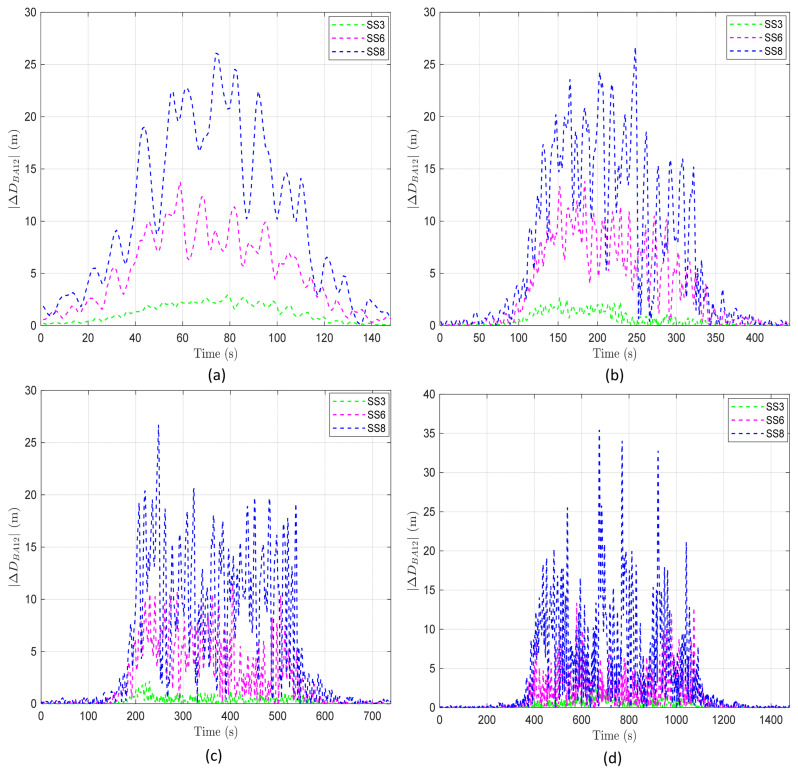
$\left|{\Delta D}_{BA12}\right|$ according to time and sea state in the overlapping region when BS1 and BS2 move in the same direction as the AUV: (**a**) Network radius of 100 m. (**b**) Network radius of 300 m. (**c**) Network radius of 500 m. (**d**) Network radius of 1000 m.

To verify this, we analyzed $\left|{\Delta D}_{BA12}\right|$ across different network radii. As shown in all figures in Figure 9, the pattern where $\left|{\Delta D}_{BA12}\right|$ exhibits higher values during the period traversing the overlapping region remains consistent. Moreover, the maximum value of $\left|{\Delta D}_{BA12}\right|$ (approximately 25 m) is also maintained regardless of the network radii, as illustrated in Figure 9a–d. This indicates that, while the modeling of BS motion results in variability in the x-, y-, and z-axis positions, the BS motion is not affected by changes in the network radius.

The statistical analysis of $\left|{\Delta D}_{BA12}\right|$ allows for more detailed examination of the impact of network radii. As shown in Table 6, an increase in network radii is associated with a decrease in both the mean and standard deviation of $\left|{\Delta D}_{BA12}\right|$. This trend is attributed to a larger network radius enhancing the proportion of time periods near the start and end of the trajectory of an AUV, where $\left|{\Delta D}_{BA12}\right|$ is close to “0” (indicating an increase in the tail of the $\left|{\Delta D}_{BA12}\right|$ pattern), thereby reducing the overall mean and standard deviation. This result confirms that the network radius influences statistical measures such as the mean and standard deviation of the variability indicator $\left|{\Delta D}_{BA12}\right|$, suggesting that an increase in network radius reduces the variability caused by BS motion.

#### 4.4.3. Analysis of Handover Decision Errors Due to Network Radii

The impact of changes in network radii on the variability indicator $\left|{\Delta D}_{BA12}\right|$, caused by BS motion, has been confirmed in Section 4.4.2. This result was analyzed to determine its effect on handover decision errors.

**Figure 10:**
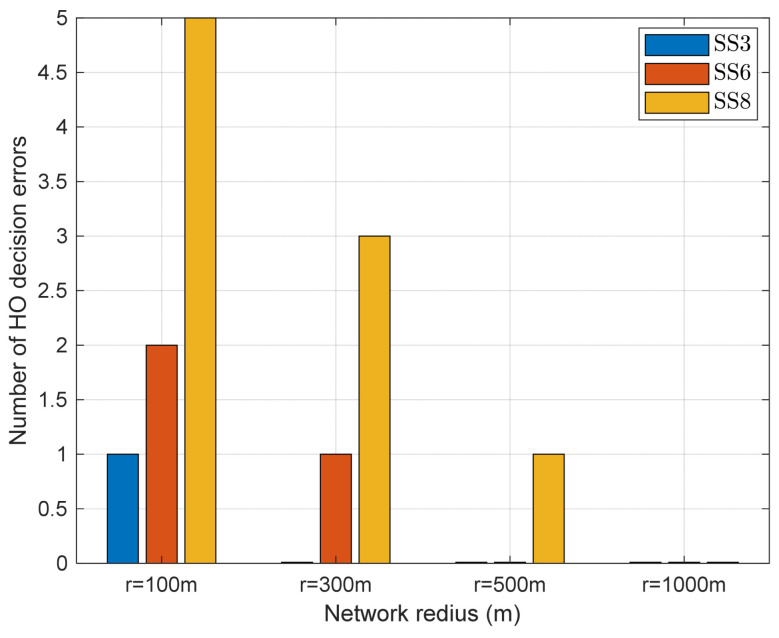
The number of handover decision errors in the overlapping region with respect to network radii when BS1 and BS2 move in the same direction as the AUV.

When the motion of BS1 and BS2 is in the same direction and the AUV passes through the overlapping region (i.e., when the handover decision error is most likely to occur), we derive the handover decision error based on the network radius and sea state code. It is expected that an increase in network radii decreases the variability in $\left|{\Delta D}_{BA12}\right|$ and, consequently, reduces handover decision errors. As illustrated in Figure 10, errors occur at SS3, SS6, and SS8 when the network radius is 100 m. Moreover, as the previous analysis has shown, the greater magnitude of the BS motion, the more errors occur, and this pattern remains the same even as the network radius changes. However, as the network radius increases, the number of errors decreases, and it becomes almost error-free at 500 m or more. These results imply that the network radius is a parameter to be considered when applying handovers for B-UWANs suffering from BS motion. This also shows that the criteria for handover decisions can be set more leniently depending on the network radius.

### 4.5. Summary and Handover Considerations

The analysis of AUV–BS distance considering BS motion was conducted from two aspects: the influence of BS motion and that of network radius, yielding the following expected simulation results:

- Considering $D_{BA}$ and $\Delta D_{BA}$, we analyzed the impact of BS motion in terms of direction and magnitude, as described in Section 4.3.1 and Section 4.3.2. It was found that the distance between the AUV and BS changes remarkably according to the direction of BS motion. In addition, the variability in AUV–BS distance is more significantly influenced by the magnitude of BS motion than its direction, emphasizing the substantial effect of the intensity of BS motion on distance fluctuations.
- We analyzed the impact of BS motion on handover by applying $D_{BA12}$ and $\Delta D_{BA12}$, as described in Section 4.3.3 and Section 4.3.4. This analysis confirmed that the motion direction of BS1 and BS2 also affects the handover as much as the motion direction between the AUV and the BS. When BS1 and BS2 move in the same direction, the variability spikes in the overlapping region. Conversely, when BS1 and BS2 move in opposite directions, the variability heightens near the start and end points of the path of the AUV. Furthermore, it was found that the symmetry of $\Delta D_{BA12}$ depends on the direction of BS motion. In particular, the analysis of $\left|{\Delta D}_{BA12}\right|$ allowed us to identify the timepoints where the impact of BS motion is most significant, and to infer the extent of variability.
- From the analysis of AUV–BS distance considering BS motion, we determined $\left|{\Delta D}_{BA12}\right|$ as an indicator to predict the likelihood of handover decision errors. By assessing how the value of $\left|{\Delta D}_{BA12}\right|$ increases during the time when handovers are executed, we can predict potential handover decision errors. The pattern of $\left|{\Delta D}_{BA12}\right|$ changes depending on the direction of BS motion, and the value of $\left|{\Delta D}_{BA12}\right|$ increases (i.e., variability increases) during the time period when passing through the overlapping region where handovers could occur, especially when the directions of BS motion are the same. Furthermore, it is observed that larger BS motions, which lead to more significant changes in the physical environment (e.g., waves and winds), can have a greater impact on the variability in AUV–BS distance. This suggests that the accuracy of deciding when to execute a handover could decrease, potentially resulting in an increase in handover decision errors.
- As described in Section 4.4, through the analysis of AUV–BS distance from the perspective of network radius, we can see that the value of the network radius also impacts $\left|{\Delta D}_{BA12}\right|$, with larger network radii resulting in a decrease in the statistical magnitude of $\left|{\Delta D}_{BA12}\right|$, thereby mitigating its influence.
- From the simulation results, it was confirmed that both BS motion and network radius are factors affecting the variability in AUV–BS distance in overlapping region. Hence, these parameters can affect handover decision errors.

The comprehensive analysis leads to the derivation of the following handover and network design considerations:

- When any underwater handover technology is designed, the BS motion needs to be considered as one of the critical factors. If the BS motion is in the same direction as the movement of an AUV, it can reduce the AUV–BS distance compared to when the BS is stationary, causing potential errors. Errors can also occur depending on the motion direction of BS1 and BS2. The direction of BS motion can alter the difference between the AUV–BS1 and AUV–BS2 distances. This situation results in the AUV incorrectly choosing a BS due to the BS motion, as it tends to connect to the network of the closest BS. However, it is hard to predict the exact direction of BS motion, so handover strategies should be developed to accommodate the worst-case scenario where BS motions are in the same direction.
- The magnitude of BS motion can also affect the extent of positional changes in the x, y, z coordinates. In other words, larger BS motions can increase the variability in the AUV–BS distance. To cope with various magnitudes of BS motion, the handover mechanism needs to be flexible. When the weather or sea conditions change, the handover triggering mechanism should be able to dynamically reflect these changes. Moreover, the threshold value of the handover criterion should not be fixed but adaptively adjusted based on the sea state to enhance network reliability.
- Since the variability induced by BS motion in the overlapping region can impact handover, it is sufficient to focus exclusively on the time duration passing through the overlapping region for handover parameters such as AUV–BS distance, without aggregating data over the entire time.
- A wider network radius extends the communication range between BSs, providing a broader margin necessary for handover decisions. This flexibility and improved reliability imply that the network radius should be set to offset the handover decision errors caused by BS motion.
- Deriving the triggering point for handover, considering the fixed positions of the BSs when an AUV moves in a certain direction (e.g., from BS1 to BS2), it is acceptable to consider triggering after passing the midpoint between the two BSs. Rather than collecting information for handover triggering from the moment a message is received from the new BS, initiating information collection for handover triggering from the point identified through analysis is more effective. This approach is efficient, as it can prevent the wasteful use of various resources, including reception power, message overhead, and processing in handover attempts. This applies equally when the network radius is large or the BS motion is minor.

## 5. Conclusions

Handover technology is necessary to guarantee seamless data service for mobile nodes such as AUVs in B-UWANs. To effectively design handover protocols for B-UWANs, it is important to address the unique challenges of underwater communication, including considerable propagation delays, constrained bandwidth, and susceptibility to various interferences that cause frequent disruptions, along with the varying positions of the BSs.

BSs are typically buoyant, floating on the water surface; thus, their positions can vary according to several factors, including currents, waves, winds, and the method of mooring. This BS motion results in variability in the distance between an AUV and a BS, which, in turn, leads to fluctuations in the received signal strength and network connectivity. We cannot precisely estimate the changes in location caused by BS motion with respect to sea conditions. Therefore, we assume that the BS is stationary when determining the AUV–BS distance. However, the actual AUV–BS distance can differ from the estimated distance due to the change in position due to the BS motion, causing errors in handover decision. Consequently, this study analyzed the impact of BS motion on handovers using the AUV–BS distance parameter, and we derived handover strategies considering the BS motion in the overlapping region.

In our simulation, we investigated how the direction and magnitude of BS motion affect the distance between the AUV and the BS. The results showed that the intensity of BS motion, quantified by the WMO sea state code, has a greater impact on distance variability than its direction. We also analyzed how BS motion influences handover decisions. Through this analysis, we derived a metric, denoted as $\left|{\Delta D}_{BA12}\right|$, to quantify the variability in AUV–BS distance caused by BS motion. When BSs move in the same direction, the variability in $\left|{\Delta D}_{BA12}\right|$ increases in the overlapping region, whereas opposite movements cause the variability in $\left|{\Delta D}_{BA12}\right|$ at the start and end points of the path of the AUV. In addition, we analyzed network radius effects on $\left|{\Delta D}_{BA12}\right|$, finding that larger radii reduce its variability.

The simulation results confirmed BS motion and network radius as critical factors in handover decision errors. Notably, errors increase in overlapping regions when the current and target BSs move in the same direction, especially with larger BS motions. Conversely, reducing the network radius lessens these errors. We developed an indicator to assess the impact of BS motion on AUV–BS distance. This comprehensive analysis provides valuable insights for designing effective underwater handovers.

Although the architecture for B-UWANs employing mobile nodes has been standardized, the development of corresponding handover technologies to support the mobility of AUVs is still in its early stages. Therefore, the insights and recommendations offered in this paper are poised to significantly contribute to the advancement of handover protocols for B-UWANs, particularly by addressing the dynamic nature of BS positions influenced by maritime conditions.

## Figures and Tables

**Table 1:** The definitions of parameters to describe the system model.

Parameters	Description
$d_{A}$	The operational depth of an AUV
$v_{A}$	The moving speed of an AUV
R	The communication range
r	The network radius of a BS on the water surface
L	The straight-line distance between the two BSs on the water surface
δ	The rate of the overlapping region between two BSs
$T_{O}$	The time during which an AUV stays in the overlapping region
$D_{BA}$	The distance between a BS and an AUV

**Table 2:** The definition of parameters related to the AUV–BS distance (the unit for all parameters in Table 2 is meters).

Parameters	Description
$D_{BA1F}$	The distance between BS1 and an AUV where BS1 is stationary
$D_{BA2F}$	The distance between BS2 and an AUV where BS2 is stationary
$D_{BA1M}$	The distance between BS1 and an AUV where BS1 is nonstationary due to BS motion
$D_{BA2M}$	The distance between BS2 and an AUV where BS2 is nonstationary due to BS motion
$D_{BA1}$	The distance between BS1 and an AUV (including both two cases)
$D_{BA2}$	The distance between a BS2 and an AUV (including both cases)
$\Delta D_{BA1}$	$\mathrm{The}\;\mathrm{difference}\;\mathrm{between}\;D_{BA1F}$$\;\mathrm{and}\;D_{BA1M}$$\;(=D_{BA1F}-D_{BA1M}$)
$\Delta D_{BA2}$	$\mathrm{The}\;\mathrm{difference}\;\mathrm{between}\;D_{BA2F}$$\;\mathrm{and}\;D_{BA2M}$$\;(=D_{BA2F}-D_{BA2M}$)
$D_{BA12F}$	$\mathrm{The}\;\mathrm{difference}\;\mathrm{between}\;D_{BA1F}$$\;\mathrm{and}\;D_{BA2F}$$\;(=D_{BA1F}-D_{BA2F}$)
$D_{BA12M}$	$\mathrm{The}\;\mathrm{difference}\;\mathrm{between}\;D_{BA1M}$$\;\mathrm{and}\;D_{BA2M}$$\;(=D_{BA1M}-D_{BA2M}$)
$D_{BA12}$	$\mathrm{The}\;\mathrm{difference}\;\mathrm{between}\;D_{BA1}$$\;\mathrm{and}\;D_{BA2}$ (including both cases)
$\Delta D_{BA12}$	$\mathrm{The}\;\mathrm{difference}\;\mathrm{between}\;D_{BA12F}$$\;\mathrm{and}\;D_{BA12M}$$\;(=D_{BA12F}-D_{BA12M}$)

**Table 3:** Simulation conditions.

Parameters	Conditions
$d_{A}$	50 m
$v_{A}$	5 knots
r	100, 300, 500, 1000 m
R	$\sqrt{r^{2}+{d_{A}}^{2}}$
δ	0.1
L	L=(1−δ)×2r
Sea State	3, 6, 8

**Table 4:** The mean and standard deviation of $\Delta D_{BA1}$ and $\Delta D_{BA2}$ according to the direction and magnitude of BS motion.

BS Motion		$\Delta D_{BA1}$ (m)						$\Delta D_{BA2}$ (m)					
		SS3		SS6		SS8		SS3		SS6		SS8	
BS1	BS2	Mean	STD	Mean	STD	Mean	STD	Mean	STD	Mean	STD	Mean	STD
F	F	0.44	0.98	1.53	4.87	2.79	10.07	−0.73	0.91	−3.55	4.63	−7.56	9.13
F	R	0.44	0.98	1.53	4.87	2.79	10.07	0.73	0.91	3.42	4.71	7.04	9.40
R	F	−0.45	0.97	−1.80	4.71	−3.88	9.36	−0.73	0.91	−3.55	4.63	−7.56	9.13
R	R	−0.45	0.97	−1.80	4.71	−3.88	9.36	0.73	0.91	3.42	4.71	7.04	9.40

**Table 5:** The mean and standard deviation of $\Delta D_{BA12}$ according to the direction and magnitude of BS motion.

BS Motion		$\Delta D_{BA12}$ @SS3 (m)		$\Delta D_{BA12}$ @SS6 (m)		$\Delta D_{BA12}$ @SS8 (m)	
BS1	BS2	Mean	STD	Mean	STD	Mean	STD
F	F	1.18	0.89	5.08	3.56	10.35	7.69
F	R	−0.28	1.69	−1.89	8.91	−4.24	17.99
R	F	0.28	1.69	1.74	8.61	3.67	16.72
R	R	−1.18	0.86	−5.23	3.65	−10.92	7.88

**Table 6:** The mean and standard deviation of $\left|{\Delta D}_{BA12}\right|$ with respect to network radii and sea state.

Network Radii (m)	$\Delta D_{BA12}$ @SS3 (m)		$\Delta D_{BA12}$ @SS6 (m)		$\Delta D_{BA12}$ @SS8 (m)	
	Mean	STD	Mean	STD	Mean	STD
100	1.18	0.85	5.08	3.56	10.35	7.69
300	0.54	0.65	3.72	3.78	7.15	7.52
500	0.46	0.55	2.44	2.90	5.66	6.36
1000	0.33	0.49	1.57	2.22	4.21	5.94

## Data Availability

The original contributions presented in this study are included in the article; further inquiries can be directed to the corresponding author.

## Appendix A

This appendix lists all of the acronyms used in this paper, as shown in Table A1.

**Table A1:** The list of acronyms used in the paper.

Acronyms
AUV: Autonomous underwater vehicle
BS: Base station
B-UWAN: Base-station-based underwater wireless acoustic network
IoT: Internet of Things
JONSWAP: Joint North Sea Wave Project
LTE: Long-term evolution
RF: Radio frequency
SS: Sea state
STD: Standard deviation
QoS: Quality of service
VHF: Very high frequency
Wi-Fi: Wireless fidelity
WMO: World Meteorological Organization

## Appendix B

This appendix describes the characteristics of the WMO sea state codes, as shown in Table A2.

**Table A2:** The characteristics of the WMO sea state codes [52].

Code	Wave Height (m)	Characteristics
0	0	Calm (glassy)
1	0 to 0.1	Calm (rippled)
2	0.1 to 0.5	Smooth
3	0.5 to 1.25	Slight
4	1.25 to 2.5	Moderate
5	2.5 to 4	Rough
6	4 to 6	Very rough
7	6 to 9	High
8	9 to 14	Very high
9	Over 14	Phenomenal
